# The Effect of Selenium Nanoparticles on the Osteogenic Differentiation of MC3T3-E1 Cells

**DOI:** 10.3390/nano11020557

**Published:** 2021-02-23

**Authors:** Sang-Cheol Lee, Na-Hyun Lee, Kapil D. Patel, Tae-Su Jang, Jonathan Campbell Knowles, Hae-Won Kim, Hae-Hyoung Lee, Jung-Hwan Lee

**Affiliations:** 1 Institute of Tissue Regeneration Engineering (ITREN), Dankook University, 119 Dandae-ro, Cheonan, Chungcheongnam-do 31116, Korea; 12201072@dankook.ac.kr (S.-C.L.); nhlee0609@dankook.ac.kr (N.-H.L.); dynamic2020@korea.ac.kr (K.D.P.); j.knowles@ucl.ac.uk (J.C.K.); kimhw@dku.edu (H.-W.K.); 2 Department of Biomaterials Science, College of Dentistry, Dankook University, 119 Dandae-ro, Cheonan, Chungcheongnam-do 31116, Korea; 3 Department of Nanobiomedical Science & BK21 PLUS NBM Global Research Center for Regenerative Medicine, Dankook University, 119 Dandae-ro, Cheonan, Chungcheongnam-do 31116, Korea; 4 Department of Materials Science and Engineering, Korea University, Seoul 02841, Korea; 5 Department of Pre-medi, College of Medicine, Dankook University, 119 Dandae-ro, Cheonan, Chungcheongnam-do 31116, Korea; jangts@dankook.ac.kr; 6 UCL Eastman-Korea Dental Medicine Innovation Centre, Dankook University, 119 Dandae-ro, Cheonan, Chungcheongnam-do 31116, Korea; 7 Division of Biomaterials and Tissue Engineering, Eastman Dental Institute, University College London, London WC1E 6HH, UK; 8 Cell & Matter Institute, Dankook University, Cheonan, Chungcheongnam-do 31116, Korea; 9 Department of Regenerative Dental Medicine, College of Dentistry, Dankook University, Cheonan, Chungcheongnam-do 31116, Korea

**Keywords:** selenium, nanoparticles, osteogenic differentiation, MC3T3-E1, reactive oxygen species

## Abstract

Reactive oxygen species (ROS) regulate various functions of cells, including cell death, viability, and differentiation, and nanoparticles influence ROS depending on their size and shape. Selenium is known to regulate various physiological functions, such as cell differentiations and anti-inflammatory functions, and plays an important role in the regulation of ROS as an antioxidant. This study aims to investigate the effect of selenium nanoparticles (SeNPs) on the differentiation of osteogenic MC3T3-E1 cells. After fabrication of SeNPs with a size of 25.3 ± 2.6 nm, and confirmation of its oxidase-like activity, SeNPs were added to MC3T3-E1 cells with or without H_2_O_2_: 5~20 μg/mL SeNPs recovered cells damaged by 200 μM H_2_O_2_ via the intracellular ROS downregulating role of SeNPs, revealed by the ROS staining assay. The increase in osteogenic maturation with SeNPs was gradually investigated by expression of osteogenic genes at 3 and 7 days, Alkaline phosphatase activity staining at 14 days, and Alizarin red S staining at 28 days. Therefore, the role of SeNPs in regulating ROS and their therapeutic effects on the differentiation of MC3T3-E1 cells were determined, leading to possible applications for bone treatment.

## 1. Introduction

Bone remodeling is mediated by osteoblasts and osteoclasts, and osteoblasts are the main cells in bone formation and are responsible for the synthesis, secretion, and mineralization of the bone matrix, which accounts for 4–6% of cell content, forming bone [1,2]. If the balance between osteoblasts and osteoclasts is abnormal, bone diseases such as osteoporosis and inflammatory bone erosion occur, threatening human health [3]. During bone regeneration, reactive oxygen species (ROS) are inevitably produced [4].

ROS is generated under the normal cellular conditions as well as a wide range of environmental stresses. Briefly, the most common types of ROS include hydroxyl radical, superoxide anion radical, and hydrogen peroxide (H_2_O_2_). Among the ROS, the H_2_O_2_ have been reported as a major source of ROS generation within the cells due to their unique features. H_2_O_2_ are nonradical molecules which have no charge, and are relatively stable and long-lived, unlike other ROS [5,6,7]. ROS regulate several cellular processes, such as proliferation, migration, and differentiation, at the physiological level [8]. However, an increase in ROS may cause cytotoxicity and is associated with conditions such as cancer, neurodegeneration, aging, and calcification of blood vessels [9,10,11,12]. Recent studies have shown that ROS can promote osteoporosis progression by inducing osteoblast death [13]. ROS are produced during bone regeneration, but in vitro tests using osteoblasts have confirmed the increase in differentiation and induction of apoptosis by ROS [14,15,16,17]. Several studies have shown an association between oxidative stress, osteogenic differentiation, and bone formation. Oxidative stress is known to damage skeletal formation and reduce osteogenic differentiation of murine osteoblast (MC3T3-E1) and bone marrow-derived stromal (M2-10B4) cell lines [17,18].

Many recent studies have focused on the growth of bone controlled by nanomaterials and the complex interactions between nanomaterials and bone cells in vivo and in vitro [19,20,21,22]. Nanomaterials, with physical properties such as size and shape, interact with cells and tissues, making them an ideal medium to accelerate tissue regeneration and improve cell proliferation [23,24,25]. Moreover, these various types of nanomaterials are widely used in fields such as diagnostics, drug delivery, and tissue engineering [26,27]. Previous studies have shown that nanoparticles play a role in promoting the differentiation of osteoblasts [28,29,30,31], and there are reports that gold nanoparticles promote the differentiation and proliferation of MC3T3-E1 cells [32]. ROS formation can be induced according to the size and shape of nanoparticles [33,34]. Since ROS not only have various functions in vivo but are key mediators of cell signaling, including cell death, viability, and differentiation, many studies have reported on the interaction between ROS and nanoparticles [35].

Selenium is one of the important trace elements in the human body that regulates various physiological functions, such as antioxidant behavior, anti-inflammatory effects, and immunity functions [36,37,38]. Selenium plays a role in relieving oxidative stress in fibroblasts subjected to heat shock [39], inhibits the cell cycle, and induces cytotoxicity and apoptosis in carcinogenic cell lines of the colon, breast, lung, or prostate [40,41,42,43,44]. Antibacterial activity was confirmed after 24 and 72 h on a coated substrate [45,46]. In addition, it is known that selenium plays an important role in the regulation of ROS by regulating the generation of ROS [47,48,49]. It has been reported that selenium has potential anticancer activity by inducing mitochondrial dysfunction and cell death [36,50]. It is also known that selenium treatment improves osteoblast differentiation of bone marrow mesenchymal stem cell (BMSC) and to protect MSCs against inhibition of osteoblast differentiation caused by H_2_O_2_ by inhibiting oxidative stress and Extracellular signal-regulated Kinase (ERK) activation [51,52]. However, the role of selenium in H_2_O_2_-induced cell-death of osteogenic cell lines is still unclear. In this study, we explored whether SeNPs protect MC3T3-E1 cells from H_2_O_2_-induced apoptosis through antioxidant activity and investigated their effect on cell differentiation.

## 2. Materials and Methods

### 2.1. Materials

Sodium selenite (Na_2_SeO_3_; Sigma-Aldrich, St. Louis, MO, USA), L-ascorbic acid (C_6_H_8_O_6_, Sigma-Aldrich, St. Louis, MO, USA), D-(+)-glucose (C_6_H_12_O_6_, Sigma, St. Louis, MO, USA), sodium hydroxide beads (NaOH, Daejung, Korea), and 1N-hydrochloric acid (HCl, Daejung, Korea) were obtained. All chemicals were of reagent grade.

### 2.2. Synthesis of Selenium Nanoparticles

Selenium nanoparticles (SeNPs) were selectively synthesized by the reduction of an aqueous solution of sodium selenite with ascorbic acid. In a typical SeNP synthesis procedure, 100 mM sodium selenite aqueous solution was mixed with 100 mM ascorbic acid, and the solution pH was adjusted to 7.1 using NaOH or HCl under rigorous magnetic stirring conditions. The resulting solution color changed to red and was centrifuged at 15,000 rpm for 30 min and washed with distilled water. The final solution was lyophilized to collect the SeNPs.

### 2.3. Characterization of SeNPs

The morphology and size, crystalline phase, chemical functional groups, surface charge, and optical properties of SeNPs were characterized by transmission electron microscopy (TEM; JEOL-7100), X-ray diffraction (XRD; Rigaku, Tokyo, Japan), Fourier transform infrared spectroscopy (FTIR; 640-IR, Varian, VIC, Australia), zeta potential (Zetasizer Nano; Malvern Instrument, Malvern, UK), and Ultraviolet-visible analysis (UV-vis, Varian Cary 100, Agilent Technologies, Paolo Alto, CA, USA), respectively.

### 2.4. Peroxidase Activity of SeNPs

The oxidase-like activity of the SeNPs was determined as previously reported [53]. Briefly, we first performed the catalytic oxidation of the peroxidase substrate 3,3′,5,5′-tetramethylbenzidine (TMB) in the presence of hydrogen peroxide (H_2_O_2_). For the experiment, varying concentrations of SeNPs (10, 20, 40, and 80 µg/mL) were dispersed in a mixture containing 350 µL of acetate buffer (0.2 M), 300 µL of TMB (0.2 M), and finally 100 µL of H_2_O_2_ (30%, 2 M). After uniform mixing, the mixture was incubated in the dark for approximately 30 min. Later, the supernatant was collected by centrifugation, and UV-visible absorption was performed in the range of 400–800 nm to confirm the oxidase-like activity of the SeNPs.

### 2.5. Cell Culture

MC3T3-E1 (ATCC, Manassas, VA, USA) cells were cultured at 37 °C in a 5% CO_2_ atmosphere in α-modified minimal essential medium without ascorbic acid (α-MEM, Welgene, Dalseogu, Daegu, Korea). Unless otherwise specified, the medium contained 10% heat-inactivated fetal bovine serum (FBS, Corning, Woodland, CA, USA), 100 U/mL penicillin, and 100 μg/mL streptomycin (Gibco, Grand Island, NY, USA). Cell medium was changed every two days. When MC3T3-E1 cells reached 80% confluence, they were detached by treatment with 0.25% trypsin Ethylenediaminetetra acetic acid (EDTA, Gibco, NY, USA) and plated for experiments. Cells used in all experiments were between 8 and 10 passages. For osteogenic differentiation, cells were seeded at a density of 6 × 10^4^ in 24-well plates. After 24 h of plating, cells were treated with osteogenic differentiation media consisting of α-MEM (α-MEM, HyClone, Logan, UT, USA) containing 10% FBS, 10 mM β-glycerophosphate (Sigma-Aldrich, St Louis, MO, USA), 10 nM dexamethasone (Sigma-Aldrich, USA), and 50 μg/mL L-ascorbic acid (Sigma-Aldrich, USA) [20].

### 2.6. Cell Viability

Cell viability was measured using a cell counting kit-8 (CCK-8, Dojindo, Kumamoto, Japan) assay. Briefly, MC3T3-E1 cells were seeded in a 96-well plate (Costar, Corning Incorporated, USA) at 1 × 10^4^ cells per well, and after 24 h, cells were treated with SeNPs (10–320 μg/mL). After 30 min of treatment with SeNPs, high oxidative stress conditions were enabled by treatment with aqueous solution of H_2_O_2_ in different concentrations (0, 100, 200, and 300 μM) for 24 h. Then, medium containing 10% CCK-8 solution was replaced in each well of the plate, followed by incubation for 2 h at 37 °C. After that, the absorbance of each well at 450 nm was recorded using a microplate reader (Thermo Fisher Varioskan^TM^ LUX, Waltham, MA, USA). Cell viability (%) was quantified by the average absorbance of the selenium treatment group/average absorbance of the control group × 100%. Cell survival was also examined by live/dead staining (0.5 μM calcein AM and 2 μM ethidium homodimer-1 solutions, Thermo Fisher, USA), and images were taken using an optical microscope (IX71, Olympus, Tokyo, Japan).

### 2.7. ROS Staining

High oxidative stress conditions were enabled by pretreatment with H_2_O_2_ for 4 h. ROS levels were measured using the Image-iT LIVE Green Reactive Oxygen Species Detection Kit (Invitrogen, Carlsbad, CA, USA). The cells were gently washed with Hanks’ Balanced Salt solution (HBSS)/Ca/Mg twice and labeled with 25 μM of 6-carboxy-2′,7′-dichlorodihydrofluorescein diacetate (carboxy-H_2_DCFDA) which is general oxidative stress indicator to cover the adherent cells for 30 min at 37 °C. The labeled cells were gently washed three times and observed using a microscope.

### 2.8. Quantitative Real Time Polymerase Chain Reaction (qRT-PCR)

MC3T3-E1 cells were cultured with or without SeNPs and H_2_O_2_ for 7 days. Total RNA was isolated using an RNA preparation kit (Geneall, Songpa-gu, Seoul, Korea) according to the manufacturer’s instructions, and the RNA concentration was measured using a Nanodrop (Thermo Fisher, USA). First-strand cDNA was developed using an RNA reverse transcription (RT) kit (Bioneer, Daeduk-gu, Daejeon, Korea) according to the manufacturer’s instructions. PCR was performed using target gene expression levels normalized to glyceraldehyde 3-phosphate dehydrogenase (GAPDH) levels. The delta cycle threshold (Ct) method was used to calculate relative levels of expression. Primer sequence is as follows: Alkaline phosphatase activity(ALP) forward 5′-ACA CCT TGA CTG TGG TTA CT-3′, reverse 5′-CCT TGT AGC CAG GCC CGT TA-3′, Osterix forward 5′-CCC TTC TCA AGC ACC AAT GG-3′, reverse 5′-AGG GTG GGT AGT CAT TTG CA-3′, GAPDH forward 5′-GAG CAT CTC CCT CAC AAT TT-3′, reverse 5′-GGG TGC AGC GAA CTT TAT-3′.

### 2.9. Alkaline Phosphatase Activity (ALP)

MC3T3-E1 cells were seeded in 24-well plates at a density of 6 × 10^4^ cells/well, and after 24 h, cells were treated with SeNPs (5–20 μg/mL). After 30 min of treatment with SeNPs, H_2_O_2_ was treated to maintain high oxidative stress conditions and osteogenic differentiation medium was treated for 14 days. ALP staining was conducted by using a staining kit (Sigma-Aldrich, USA). The cells were washed with phosphate buffer solution (PBS, Tech and Innovation, Chuncheon, Korea) twice and fixed with 4% paraformaldehyde (PFA) for 30 min at room temperature. The fixed cells were washed with PBS three times and stained with ALP solution dissolved in distilled water for 1 h at 37 °C. Stained cells were washed with PBS three times and observed using an optical microscope. After staining, quantification was performed using ImageJ software (Bethesda, MD, USA).

### 2.10. Alizarin Red S Staining (ARS)

Alizarin red S (ARS, Sigma-Aldrich, USA) staining was performed to determine MC3T3-E1 mineralization. MC3T3-E1 cells were seeded in 24-well plates at a density of 6 × 10^4^ cells/well in osteogenic differentiation medium for 28 days. The cells were washed twice with PBS and then fixed with 4% PFA for 30 min. The fixed cells were also washed 3 times with PBS and subsequently stained with 40 mM ARS solution for 10 min. The remaining solution was washed out 5 times with distilled water and observed using a microscope. The stained calcium deposits were dissolved in 10% (*w/v*) cetylpyridinium chloride (CPC, Sigma-Aldrich, MO, USA) solution on a rocking shaker. After 1 h, the eluted solutions were transferred to 96-well plates and measured at 562 nm using a microplate reader.

### 2.11. Statistical Analysis

Statistical significance between groups was evaluated by one-way analysis of variance (ANOVA) followed by Dunnett’s multiple comparisons tests or two-way ANOVA followed by Dunnett’s multiple comparisons tests. GraphPad Prism 8 software (San Diego, CA, USA) was used.

## 3. Results and Discussion

### 3.1. Characterization of SeNPs

**Figure 1:**
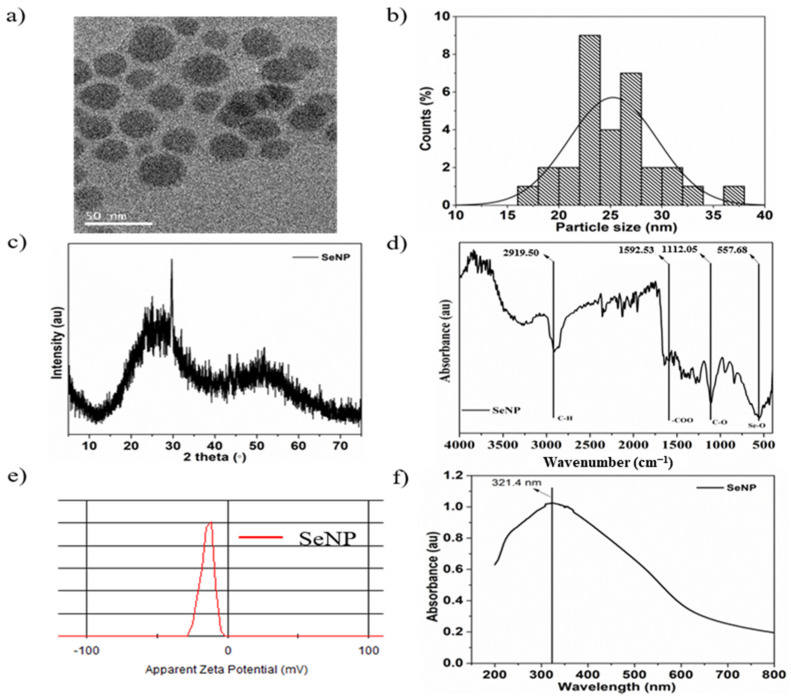
Characterization of selenium nanoparticles (SeNPs): (**a**) Transmission electron microscopy (TEM) image of SeNPs, (**b**) particle size distribution, (**c**) X-ray diffraction (XRD) pattern, (**d**) Fourier transform infrared (FTIR) spectrum, (**e**) ξ-potential, and (**f**) UV-visible spectrum of SeNPs in distilled water.

The morphology and shape of the sol-gel synthesized SeNPs are shown in the transmission electron microscopy (TEM) image in Figure 1a. The SeNPs were spherical in shape with a size of 25.3 ± 2.6 nm, and the particle size distribution was analyzed by ImageJ software. Figure 1b shows the particle size distribution histogram from 18 to 32 nm. The XRD pattern of SeNPs is shown in Figure 1c and exhibits two broad peaks at 2θ = 20–30° and 45–55°, demonstrating the crystalline and amorphous nature of the particles [54]. The FTIR spectra of the SeNPs showed four main peaks, as shown in Figure 1d. The sharp and intense peak at 2919.5 cm^−1^ corresponds to the –CH group, the peak at 1592.53 cm^−1^ corresponds to the –COO group, and the peaks at 1112.05 and 557.68 cm^−1^ correspond to the –CO and Se-O groups, respectively [55,56]. Furthermore, the surface charge of the SeNPs was estimated to be −13.9 mVas, as shown in Figure 1e, which is mainly due to the presence of –COO, –CO, and –OH groups on the surface, as verified by FTIR analysis. Higher zeta potentials, either negative or positive, provide stable particle colloidal suspensions. The optical properties of the SeNPs were analyzed by UV-visible spectroscopy. The absorption spectra of the SeNPs are depicted in Figure 1f. The SeNPs exhibit a broad absorption peak at 321.4 nm [57].

### 3.2. Oxidase-Like Activity

**Figure 2:**
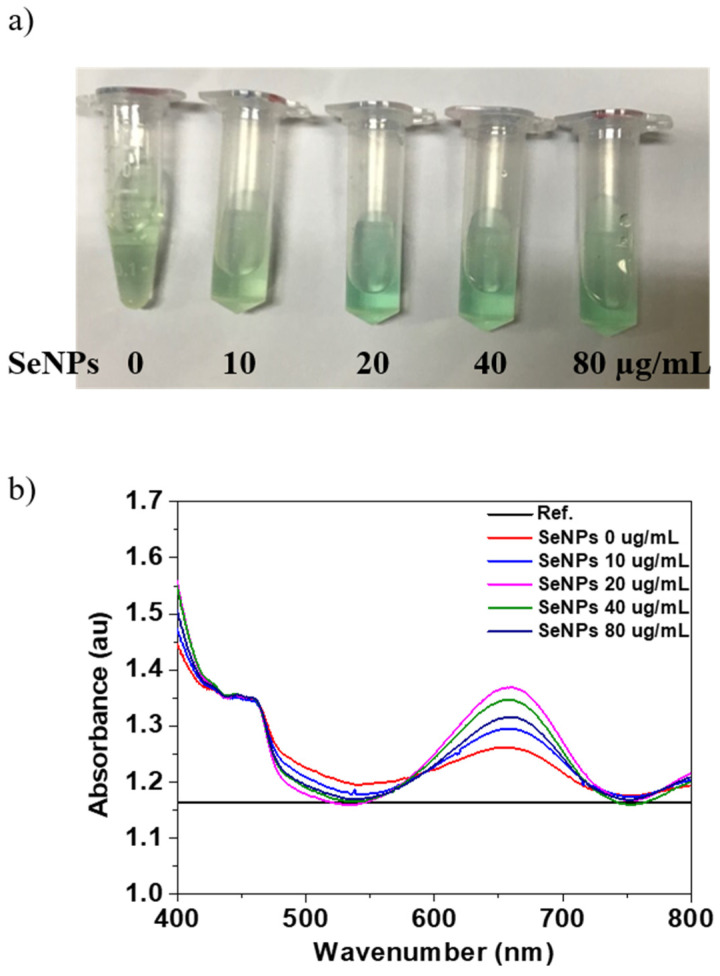
Determination of oxidase properties of different concentrations of SeNPs: (**a**) The color evaluation of TMB oxidation with SeNP concentrations of 0, 10, 20, 40, and 80 µg/mL, (**b**) the UV-visible absorption spectra of TMB (before reaction as Reference) and after oxidation for 30 min with and without SeNPs.

The oxidase-like activity of the SeNPs was performed using the TMB assay. Figure 2a shows the optical image of the TMB and SeNPs with concentration from 10 to 80 µg/mL in TMB and produces typical light blue color after 30 min of reaction. The changing of color of SeNPs in TMB to blue in the presence of H_2_O_2_ is the confirmation of oxidase-like activity of SeNPs. After confirmation of oxidase-like activity of SeNPs, we performed the UV-visible absorption for the quantification, and the maximum absorption peak of the oxidized TMB products was observed at 652 nm. The UV-visible absorption spectrum of the TMB solutions before (Reference) and after reaction (0 µg/mL) and SeNPs in TMB solutions after reaction of 30 min is shown in Figure 2a. The oxidized products with TMB solution in the presence of SeNPs shows higher absorption peak intensity at 652 nm, which was quantified. The quantitative analysis of the absorption values of the TMB and SeNPs in TMB solution are shown in Figure 2b. The intensity of the absorption peak at 652 nm increases with increasing the SeNPs concentrations, which confirmed the oxidation of TMB and intrinsic oxidase-like activity of the prepared SeNPs. Moreover, it is common to increase the oxidase activity with increasing the concentration of the nanoparticles, as reported in other reports [58]. It has also been reported that the smaller the nanoparticles’ size, the higher the oxidase activity because the smaller size would expose more active sites due to the high surface-to-volume ratio [59]. Overall, our results suggest that 40 µg/mL concentration of SeNPs is the optimal concentration to achieve effective oxidase activity.

### 3.3. Effect of Selenium Nanoparticles on Cell Viability in MC3T3-E1-Induced ROS Conditions

**Figure 3:**
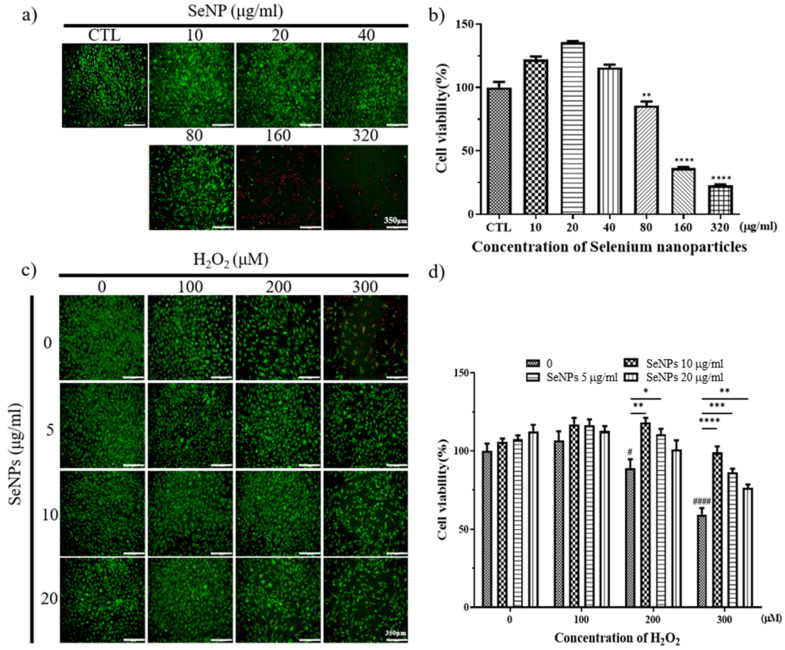
Cell viability and proliferation were determined by live (green) and dead (red) and CCK-8 assays. After cell seeding for 24 h, MC3T3-E1 cells were analyzed using CCK-8 solution and stained using live and dead staining to evaluate cell viability. (**a**) Live and dead staining following treatment with various SeNP concentrations. (**b**) The relative cell viability of MC3T3-E1 cells cultured in different concentrations of SeNPs. (**c**) Live and dead staining following treatment with various SeNP concentrations and H_2_O_2_. (**d**) Cell viability of MC3T3-E1 cells treated with various concentrations of SeNPs and H_2_O_2_. The statistical significance of (b) was calculated using one-way analysis of variance (ANOVA), and (d) was calculated using two-way ANOVA followed by a two-sided Dunnett’s multiple comparison test compared to control (CTL) (scale bar = 350 μm). * Represents *p* < 0.05, ** *p* < 0.01, *** *p* < 0.001, **** *p* < 0.0001, ^#^ is compared with SeNPs and H_2_O_2_-untreated groups. ^#^ Represents *p* < 0.05, ^####^ *p* < 0.0001; *n* = 4.

Thirty-two milligrams of SeNPs were quantified and dissolved in PBS. After that, the selenium stock was diluted to the highest concentration of 320 μg/mL and used to treat MC3T3-E1 cells, followed by serial dilution by 1/2. As a result of treatment with SeNPs, MC3T3-E1 cell viability was shown at a concentration of 40 μg/mL or less, confirmed by Live/Dead staining (Figure 3a) and CCK-8 assay (Figure 3b), respectively. In the case of treatment of MC3T3-E1 cells with hydrogen peroxide, Live/Dead staining images showed that cells survived in H_2_O_2_ concentrations of 400 μM or less (Figure 3c). As a result of treatment with SeNPs and H_2_O_2_ together, cell viability increased compared to the untreated group when selenium was applied, and the SeNP-treated group showed an approximately 17.24–39.58% increase in cell viability compared to the SeNP-untreated group, when 300 μM H_2_O_2_ was treated (Figure 3d). This confirmed the cell viability at a relatively higher selenium concentration compared to previous studies that treated MC3T3-E1 cells with selenium at 0–800 ng/mL [60]. The highest concentration of 20 μg/mL SeNPs and 200 μM H_2_O_2_ was used for further experiments, and we analyzed the effect of SeNPs on ROS in H_2_O_2_.

### 3.4. ROS Staining

**Figure 4:**
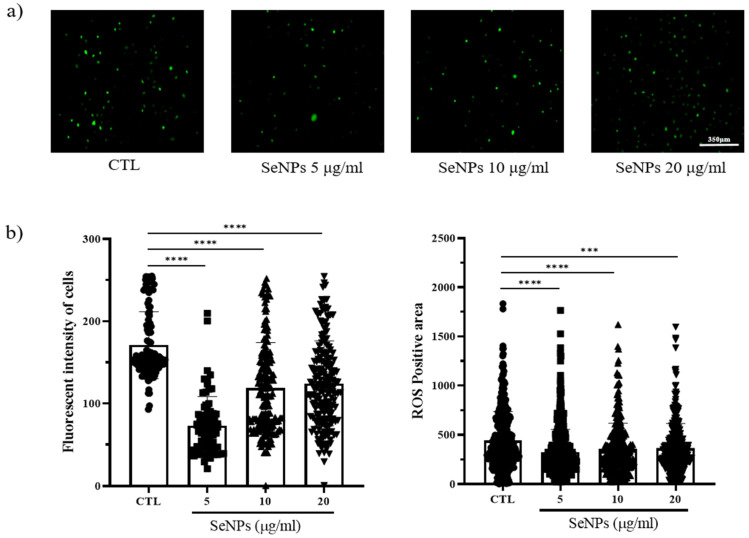
MC3T3-E1 cells were exposed to 400 μM H_2_O_2_ for oxidative stress and then recovered by culturing in medium with or without SeNPs. High oxidative stress conditions were enabled by pretreatment with H_2_O_2_ for 4 h. (**a**) SeNP treatment resulted in reduced levels of reactive oxygen species (ROS). (**b**) The fluorescence intensity of cells and ROS-positive areas was measured using ImageJ. Statistical significance was calculated using one-way ANOVA followed by a two-sided Dunnett post hoc test compared to CTL (scale bar = 350 μm). **** Represents *p* < 0.0001; n = 5.

Because ROS generation is mostly governed by mitochondria, loss of the mitochondrial membrane triggers ROS generation, and increased ROS production leads to further mitochondrial disruption. We thus examined whether SeNP treatment affected ROS production. To measure ROS production, we used 5-(and-6)-carboxy-29,79-dichlorodihydrofluorescein diacetate (carboxy H_2_DCFDA) after SeNP and 400 μM H_2_O_2_ treatment. As shown in Figure 4a, control cells showed a large number of cells stained with fluorescence. In contrast, the cells treated with 5 μg/mL SeNPs showed weak fluorescence, indicating that 5 μg/mL SeNPs efficiently controlled ROS. Furthermore, it has been suggested that ROS may affect several cellular activities. Additionally, Figure 4b shows the results obtained by analyzing the intensity and positive area of the staining value using ImageJ. As a result of the intensity analysis of fluorescently stained cells, the SeNP-treated group showed lower intensity than the untreated group, and the highest decrease was observed at 5 μg/mL SeNPs. This result suggests that selenium nanoparticles can be involved in various activities of cells by regulating ROS, in addition to previous studies showing that SeNPs act as antioxidants [61,62].

### 3.5. Effect of Selenium Nanoparticles on the Expression of Osteogenic Genes Determined by qRT-PCR

**Figure 5:**
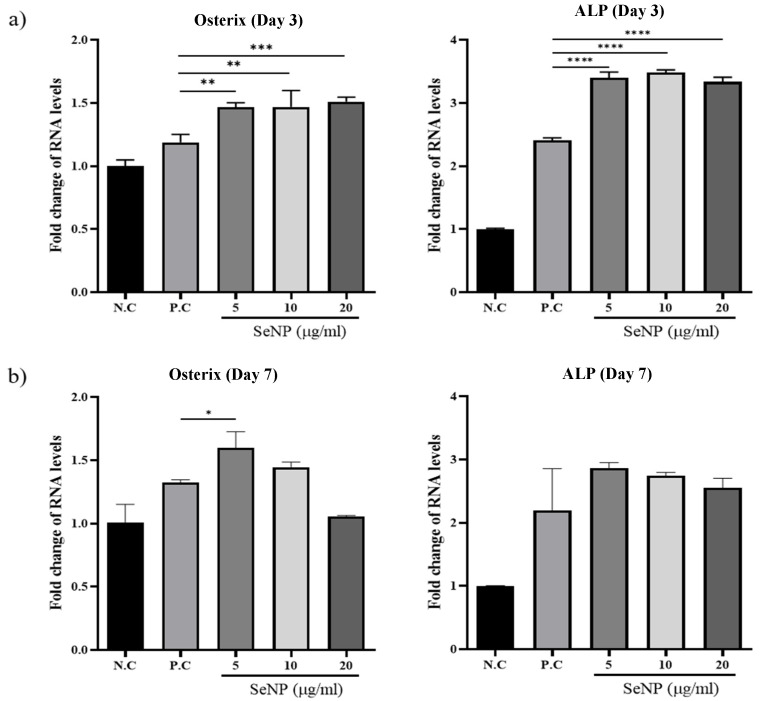
The effect of SeNPs on the expression of osteogenic genes through qRT-PCR. The relative expression levels of target genes normalized to GAPDH were calculated using the delta cycle threshold (Ct) method. The figure shows the relative expression of multiple genes relative to gene expression in the negative control treatment cells. (**a**) Results of qRT-PCR analysis of osteogenic markers on the third day after treatment with osteogenic differentiation media. In the group treated with selenium, the activities of osterix and ALP were higher than those in the group not treated with selenium. (**b**) Results of qRT-PCR analysis on the seventh day after treatment with osteogenic differentiation media. The selenium-treated group showed higher osterix and ALP activity than the selenium-treated group, and the highest gene expression was confirmed at 5 μg/mL. Statistical significance was calculated using one-way ANOVA followed by a two-sided Dunnett post hoc test compared to CTL (N.C: Negative control, P.C: Positive control). * Represents *p* < 0.05, ** *p* < 0.01, *** *p* < 0.001, **** *p* < 0.0001; *n* = 3.

qRT-PCR was used to investigate the expression levels of osterix, one of the major osteoblast transcription factors in bone formation [63], and ALP, one of the most reliable markers for osteogenic differentiation produced by osteogenic cells [64,65,66], to determine the effect of SeNPs on the expression levels of MC3T3-E1 cells. Figure 5a shows that treatment with SeNPs for 3 days resulted in an increase in the expression of the osteogenic genes analyzed. For osterix, the value of the negative control group was 1.00 ± 0.05, the positive control was 1.19 ± 0.06, SeNPs at 5 μg/mL was 1.47 ± 0.03, SeNPs at 10 μg/mL was 1.47 ± 0.13, and SeNPs at 20 μg/mL was 1.51 ± 0.03. In the case of ALP, the value of the negative control group was 1.00 ± 0.01, the positive control was 2.41 ± 0.04, SeNPs at 5 μg/mL was 3.40 ± 0.09, SeNPs at 10 μg/mL was 3.48 ± 0.04, and SeNPs at 20 μg/mL was 3.33 ± 0.07. Figure 5b shows the results after treatment with SeNPs for 7 days. In the case of osterix, the value of the negative control group was 1.00 ± 0.14, the positive control was 1.32 ± 0.02, SeNPs at 5 μg/mL was 1.60 ± 0.13, SeNPs at 10 μg/mL was 1.45 ± 0.04, and SeNPs at 20 μg/mL was 1.05 ± 0.01. According to the ALP expression results, the value of the negative control group was 1.00 ± 0.01, the positive control was 2.19 ± 0.66, SeNPs at 5 μg/mL was 2.86 ± 0.08, SeNPs at 10 μg/mL was 2.75 ± 0.05, and SeNPs at 20 μg/mL was 2.55 ± 0.15.

### 3.6. Effect of Selenium Nanoparticles on Cell Differentiation

**Figure 6:**
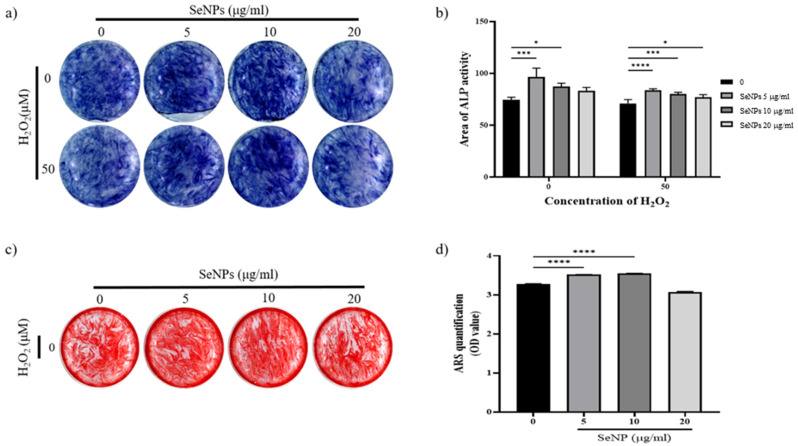
Effect of SeNPs on ALP activity and mineralization in MC3T3-E1 cells. (**a**) Results of ALP staining of cells on day 14 after treatment with different concentrations of SeNPs. Higher ALP activity was shown in the 5–10 μg/mL SeNP-treated group than in the SeNP nontreated group. (**b**) ALP quantitative graph. Both H_2_O_2_-treated and untreated groups showed high ALP activity at SeNP concentrations of 5–10 μg/mL. (**c**) The formation of calcium deposits is indicated by ARS staining. After treatment with different concentrations of SeNPs, cells were stained with ARS 28 days after treatment with osteogenic differentiation medium. Higher calcium deposition was observed in the group treated with 5 μg/mL selenium and 10 μg/mL selenium without H_2_O_2_. (**d**) Results of quantification of ARS staining using CPC extraction. Statistical significance was calculated using one-way ANOVA followed by a two-sided Dunnett post hoc test compared to CTL. * Represents *p* < 0.05, *** *p* < 0.001, **** *p* < 0.0001.

To assess whether SeNPs were effective for the differentiation of MC3T3-E1 cells, ALP activity was measured at 14 days. Figure 6a shows the ALP staining data of MC3T3-E1 cells treated with SeNPs for 14 days. ALP staining was performed for 14 days after cells were treated with osteogenic differentiation media. The results of ALP staining in 5–10 μg/mL SeNP-treated cells were higher than those in positive control cells. In addition, even at 50 μM H_2_O_2_, the staining was higher in the 5–10 μg/mL SeNP-treated group than in the SeNP-untreated group. Figure 6b showed quantification of ALP staining. Both H_2_O_2_-treated and untreated groups showed high ALP activity at SeNP concentrations of 5–10 μg/mL. Based on these results, it was confirmed that SeNPs express early osteogenic markers such as ALP in the H_2_O_2_ environment. As a result of ALP staining, higher ALP activity was confirmed in H_2_O_2_ untreated group. Therefore, the mineralization of MC3T3-E1 cells were investigated through ARS staining in the H_2_O_2_-untreated group. Another osteoblast differentiation marker, ARS staining, was used to evaluate the level of differentiation. The effects of SeNPs on the development of calcium deposits in MC3T3-E1 cells were analyzed by ARS staining. Figure 6c shows that SeNPs promoted the mineralization function of MC3T3-E1 cells. Cells treated with osteogenic differentiation media (ODM) and 5–10 μg/mL SeNPs showed higher staining than the group treated only with ODM, and the group treated with 50 μM H_2_O_2_ showed lower staining than the selenium-treated group. Figure 6d shows a graph quantified by adding CPC solution to the stained well after ARS staining. In the 5–10 μg/mL SeNP-treated group showed higher mineralization than the SeNPs untreated group. Through these results, it was confirmed that SeNPs at a concentration of 5–10 μg/mL promote the differentiation of MC3T3-E1 cells. In addition to previous studies reporting improved cell adhesion and osteoblast function [36], SeNPs protect against ROS-induced damage and are related to cell growth and differentiation regulation [67,68,69], confirming that they also affect the differentiation of cells.

## 4. Conclusions

**Figure 7:**
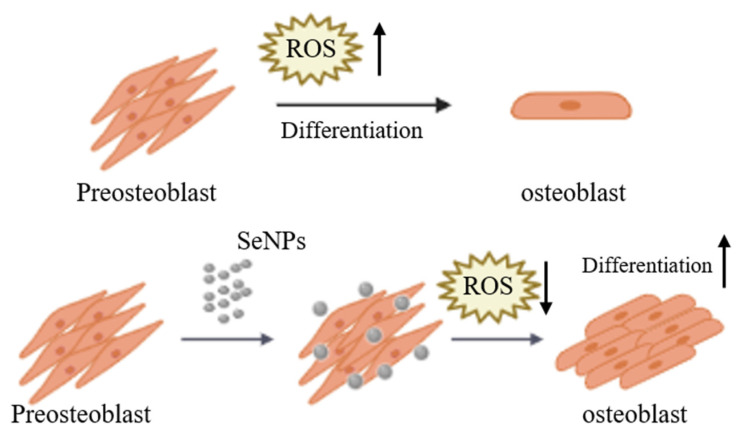
Schematic diagram of MC3T3-E1 cells cultured and treated with SeNPs for osteogenic differentiation. ROS increase during the differentiation of pre-osteoblasts into osteoblasts. SeNP treatment regulates ROS to protect cells from ROS and is also involved in cell differentiation.

The purpose of this study was to investigate the ROS regulation ability and efficiency of SeNPs in MC3T3-E1 cells for ROS produced during the cell differentiation (Figure 7). In this study, as a result of analyzing the cell viability after treatment of MC3T3-E1 cells with SeNP and H_2_O_2_ together, cell viability was confirmed at a SeNP concentration of 20 μg/mL or less and H_2_O_2_ concentration of 200 μM or less. In addition, it was confirmed that SeNP can control ROS and protect cells by treating H_2_O_2_. As a result of qRT-PCR to investigate the expression level of the osteogenic differentiation marker, it was confirmed that gene expression increased in the SeNP (5–10 μg/mL) treatment group compared to the control group 3 and 7 days after treatment with the osteogenic differentiation medium. We analyzed the effect of SeNP on the differentiation of MC3T3-E1 cells at high free radical concentrations. As a result of ALP staining 14 days after the osteogenic differentiation medium treatment, it was confirmed that ALP activity was higher in the 5–10 μg/mL SeNP-treated group and the untreated group. As a result of ALP activity analysis, higher ALP activity was confirmed in the H_2_O_2_ untreated group than in the treated group, and the degree of calcification of MC3T3-E1 cells according to SeNP treatment proceeded only in the H_2_O_2_ untreated group. ARS staining was performed 28 days after treatment with bone differentiation medium. In the H_2_O_2_ untreated group, the SeNP-treated group showed higher staining at a concentration of 5–10 μg/mL compared to the untreated group. Based on these results, the treatment of SeNPs seems to have an effect on early differentiation but not on late differentiation. These results suggest that SeNPs may affect the differentiation of MC3T3-E1 cells. In addition, previous studies have shown that SeNP protects LPS-treated MC3T3-E1 cells from death through regulation of the phosphatidylinositol 3-kinase (PI3K)/protein kinase B (AKT) signaling pathway and improves cell adhesion and osteoblast function. However, in this paper, we confirmed the potential as a bone therapy through studies on whether SeNP, which has not been studied previously, modulates ROS and affects the differentiation of MC3T3-E1 cells. 

## Data Availability

Not applicable.

## References

[1] Yang J.Y., Jung J.Y., Cho S.W., Choi H.J., Kim S.W., Kim S.Y., Kim H.J., Jang C.H., Lee M.G., Han J. (2009). Chloride intracellular channel 1 regulates osteoblast differentiation. Bone.

[2] Liu D., Yi C., Zhang D., Zhang J., Yang M. (2010). Inhibition of proliferation and differentiation of mesenchymal stem cells by carboxylated carbon nanotubes. ACS Nano.

[3] Shah M., Kola B., Bataveljic A., Arnett T.R., Viollet B., Saxon L., Korbonits M., Chenu C. (2010). AMP-activated protein kinase (AMPK) activation regulates in vitro bone formation and bone mass. Bone.

[4] Callaway D.A., Jiang J.X. (2015). Reactive oxygen species and oxidative stress in osteoclastogenesis, skeletal aging and bone diseases. J. Bone Miner. Metab..

[5] Bienert G.P., Moller A.L., Kristiansen K.A., Schulz A., Moller I.M., Schjoerring J.K., Jahn T.P. (2007). Specific aquaporins facilitate the diffusion of hydrogen peroxide across membranes. J. Biol. Chem..

[6] He L., He T., Farrar S., Ji L., Liu T., Ma X. (2017). Antioxidants Maintain Cellular Redox Homeostasis by Elimination of Reactive Oxygen Species. Cell. Physiol. Biochem..

[7] Sharma P., Jha A.B., Dubey R.S., Pessarakli M. (2012). Reactive oxygen species, oxidative damage, and antioxidative defense mechanism in plants under stressful conditions. J. Bot..

[8] Droge W. (2002). Free radicals in the physiological control of cell function. Physiol. Rev..

[9] Ahamed M., Akhtar M.J., Khan M., Alhadlaq H.A., Alshamsan A. (2020). Barium Titanate (BaTiO_3_) Nanoparticles Exert Cytotoxicity through Oxidative Stress in Human Lung Carcinoma (A549) Cells. Nanomaterials.

[10] Shigenaga M.K., Hagen T.M., Ames B.N. (1994). Oxidative damage and mitochondrial decay in aging. Proc. Natl. Acad. Sci. USA.

[11] Mody N., Parhami F., Sarafian T.A., Demer L.L. (2001). Oxidative stress modulates osteoblastic differentiation of vascular and bone cells. Free Radic. Biol. Med..

[12] Floyd R.A. (1990). Role of oxygen free radicals in carcinogenesis and brain ischemia. FASEB J..

[13] Liu Y., Wang C., Wang G., Sun Y., Deng Z., Chen L., Chen K., Tickner J., Kenny J., Song D. (2019). Loureirin B suppresses RANKL-induced osteoclastogenesis and ovariectomized osteoporosis via attenuating NFATc1 and ROS activities. Theranostics.

[14] Wang X., Wu T.T., Jiang L., Rong D., Zhu Y.Q. (2017). Deferoxamine-Induced Migration and Odontoblast Differentiation via ROS-Dependent Autophagy in Dental Pulp Stem Cells. Cell. Physiol. Biochem..

[15] Arakaki N., Yamashita A., Niimi S., Yamazaki T. (2013). Involvement of reactive oxygen species in osteoblastic differentiation of MC3T3-E1 cells accompanied by mitochondrial morphological dynamics. Biomed. Res..

[16] Domazetovic V., Marcucci G., Iantomasi T., Brandi M.L., Vincenzini M.T. (2017). Oxidative stress in bone remodeling: Role of antioxidants. Clin. Cases Miner. Bone Metab..

[17] Atashi F., Modarressi A., Pepper M.S. (2015). The role of reactive oxygen species in mesenchymal stem cell adipogenic and osteogenic differentiation: A review. Stem Cells Dev..

[18] Li Y., Chen G., He Y., Zhang X., Zeng B., Wang C., Yi C., Yu D. (2019). Ebselen rescues oxidative-stress-suppressed osteogenic differentiation of bone-marrow-derived mesenchymal stem cells via an antioxidant effect and the PI3K/Akt pathway. J. Trace Elem. Med. Biol..

[19] Wu Z., Meng Z., Wu Q., Zeng D., Guo Z., Yao J., Bian Y., Gu Y., Cheng S., Peng L. (2020). Biomimetic and osteogenic 3D silk fibroin composite scaffolds with nano MgO and mineralized hydroxyapatite for bone regeneration. J. Tissue Eng..

[20] Yang X.-F., Chen Y., Yang F., He F.-M., Zhao S.-F. (2009). Enhanced initial adhesion of osteoblast-like cells on an anatase-structured titania surface formed by H_2_O_2_/HCl solution and heat treatment. Dent. Mater..

[21] Bedair T.M., Lee C.K., Kim D.S., Baek S.W., Bedair H.M., Joshi H.P., Choi U.Y., Park K.H., Park W., Han I. (2020). Magnesium hydroxide-incorporated PLGA composite attenuates inflammation and promotes BMP2-induced bone formation in spinal fusion. J. Tissue Eng..

[22] Fatokun A.A., Stone T.W., Smith R.A. (2008). Responses of differentiated MC3T3-E1 osteoblast-like cells to reactive oxygen species. Eur. J. Pharmacol..

[23] Singh R.K., Knowles J.C., Kim H.W. (2019). Advances in nanoparticle development for improved therapeutics delivery: Nanoscale topographical aspect. J. Tissue Eng..

[24] Qin D., Zhang H., Zhang H., Sun T., Zhao H., Lee W.H. (2019). Anti-osteoporosis effects of osteoking via reducing reactive oxygen species. J. Ethnopharmacol..

[25] Takanche J.S., Kim J.E., Han S.H., Yi H.K. (2020). Effect of gomisin A on osteoblast differentiation in high glucose-mediated oxidative stress. Phytomedicine.

[26] Mazzola L. (2003). Commercializing nanotechnology. Nat. Biotechnol..

[27] Paull R., Wolfe J., Hebert P., Sinkula M. (2003). Investing in nanotechnology. Nat. Biotechnol..

[28] Beck G.R., Ha S.W., Camalier C.E., Yamaguchi M., Li Y., Lee J.K., Weitzmann M.N. (2012). Bioactive silica-based nanoparticles stimulate bone-forming osteoblasts, suppress bone-resorbing osteoclasts, and enhance bone mineral density in vivo. Nanomedicine.

[29] Ha S.-W., Weitzmann M.N., Beck G.R. (2014). Bioactive silica nanoparticles promote osteoblast differentiation through stimulation of autophagy and direct association with LC3 and p62. ACS Nano.

[30] Liu Y., Zheng Z., Zara J.N., Hsu C., Soofer D.E., Lee K.S., Siu R.K., Miller L.S., Zhang X., Carpenter D. (2012). The antimicrobial and osteoinductive properties of silver nanoparticle/poly (DL-lactic-co-glycolic acid)-coated stainless steel. Biomaterials.

[31] Weitzmann M.N., Ha S.W., Vikulina T., Roser-Page S., Lee J.K., Beck G.R. (2015). Bioactive silica nanoparticles reverse age-associated bone loss in mice. Nanomedicine.

[32] Yao Y., Shi X., Chen F. (2014). The effect of gold nanoparticles on the proliferation and differentiation of murine osteoblast: A study of MC3T3-E1 cells in vitro. J. Nanosci. Nanotechnol..

[33] Akter M., Sikder M.T., Rahman M.M., Ullah A., Hossain K.F.B., Banik S., Hosokawa T., Saito T., Kurasaki M. (2018). A systematic review on silver nanoparticles-induced cytotoxicity: Physicochemical properties and perspectives. J. Adv. Res..

[34] Rajoria S., Rani S., Chaudhari D., Jain S., Gupta U. (2019). Glycine-Poly-L-Lactic Acid Copolymeric Nanoparticles for the Efficient Delivery of Bortezomib. Pharm. Res..

[35] Yu Z., Li Q., Wang J., Yu Y., Wang Y., Zhou Q., Li P. (2020). Reactive Oxygen Species-Related Nanoparticle Toxicity in the Biomedical Field. Nanoscale Res. Lett..

[36] Karahaliloglu Z., Kilicay E. (2020). In vitro evaluation of bone cements impregnated with selenium nanoparticles stabilized by phosphatidylcholine (PC) for application in bone. J. Biomater. Appl..

[37] Vera P., Canellas E., Nerín C. (2018). New antioxidant multilayer packaging with nanoselenium to enhance the shelf-life of market food products. Nanomaterials.

[38] Dorazilová J., Muchová J., Šmerková K., Kočiová S., Diviš P., Kopel P., Veselý R., Pavliňáková V., Adam V., Vojtová L. (2020). Synergistic Effect of Chitosan and Selenium Nanoparticles on Biodegradation and Antibacterial Properties of Collagenous Scaffolds Designed for Infected Burn Wounds. Nanomaterials.

[39] Yuan B., Webster T.J., Roy A.K. (2016). Cytoprotective effects of cerium and selenium nanoparticles on heat-shocked human dermal fibroblasts: An in vitro evaluation. Int. J. Nanomed..

[40] Badr D.M., Hafez H.F., Agha A.M., Shouman S.A. (2016). The Combination of alpha-Tocopheryl Succinate and Sodium Selenite on Breast Cancer: A Merit or a Demerit?. Oxid. Med. Cell. Longev..

[41] Berggren M., Sittadjody S., Song Z., Samira J.L., Burd R., Meuillet E.J. (2009). Sodium selenite increases the activity of the tumor suppressor protein, PTEN, in DU-145 prostate cancer cells. Nutr. Cancer.

[42] Kieliszek M., Lipinski B., Blazejak S. (2017). Application of Sodium Selenite in the Prevention and Treatment of Cancers. Cells.

[43] Schroterova L., Kralova V., Voracova A., Haskova P., Rudolf E., Cervinka M. (2009). Antiproliferative effects of selenium compounds in colon cancer cells: Comparison of different cytotoxicity assays. Toxicol. In Vitro.

[44] Pang K.L., Chin K.Y. (2019). Emerging Anticancer Potentials of Selenium on Osteosarcoma. Int. J. Mol. Sci..

[45] Tran P.A., Webster T.J. (2013). Antimicrobial selenium nanoparticle coatings on polymeric medical devices. Nanotechnology.

[46] Wang Q., Webster T.J. (2012). Nanostructured selenium for preventing biofilm formation on polycarbonate medical devices. J. Biomed. Mater. Res. Part A.

[47] Arteel G.E., Sies H. (2001). The biochemistry of selenium and the glutathione system. Environ. Toxicol. Pharmacol..

[48] Cardoso B.R., Roberts B.R., Bush A.I., Hare D.J. (2015). Selenium, selenoproteins and neurodegenerative diseases. Metallomics.

[49] Stolzoff M., Webster T.J. (2016). Reducing bone cancer cell functions using selenium nanocomposites. J. Biomed. Mater. Res. Part A.

[50] Rayman M.P. (2000). The importance of selenium to human health. Lancet.

[51] Li C., Wang Q., Gu X., Kang Y., Zhang Y., Hu Y., Li T., Jin H., Deng G., Wang Q. (2019). Porous Se@SiO_2_ nanocomposite promotes migration and osteogenic differentiation of rat bone marrow mesenchymal stem cell to accelerate bone fracture healing in a rat model. Int. J. Nanomed..

[52] Liu H., Bian W., Liu S., Huang K. (2012). Selenium protects bone marrow stromal cells against hydrogen peroxide-induced inhibition of osteoblastic differentiation by suppressing oxidative stress and ERK signaling pathway. Biol. Trace Elem. Res..

[53] Guo L., Huang K., Liu H. (2016). Biocompatibility selenium nanoparticles with an intrinsic oxidase-like activity. J. Nanopart. Res..

[54] Xia Y., Tang G., Guo M., Xu T., Chen H., Lin Z., Li Y., Chen Y., Zhu B., Liu H. (2020). Silencing KLK12 expression via RGDfC-decorated selenium nanoparticles for the treatment of colorectal cancer in vitro and in vivo. Mater. Sci. Eng. C Mater. Biol. Appl..

[55] Tugarova A.V., Mamchenkova P.V., Dyatlova Y.A., Kamnev A.A. (2018). FTIR and Raman spectroscopic studies of selenium nanoparticles synthesised by the bacterium Azospirillum thiophilum. Spectrochim. Acta Part A Mol. Biomol. Spectrosc..

[56] Gunti L., Dass R.S., Kalagatur N.K. (2019). Phytofabrication of Selenium Nanoparticles From Emblica officinalis Fruit Extract and Exploring Its Biopotential Applications: Antioxidant, Antimicrobial, and Biocompatibility. Front. Microbiol..

[57] Wadhwani S., Gorain M., Banerjee P., Shedbalkar U., Singh R., Kundu G., Chopade B. (2017). Green synthesis of selenium nanoparticles using *Acinetobacter* sp. SW30: Optimization, characterization and its anticancer activity in breast cancer cells. Int. J. Nanomed..

[58] Cao H., Xiao J., Liu H. (2019). Enhanced oxidase-like activity of selenium nanoparticles stabilized by chitosan and application in a facile colorimetric assay for mercury (II). Biochem. Eng. J..

[59] Wu X., Zhao G., He Y., Wang W., Yang C.S., Zhang J. (2019). Pharmacological mechanisms of the anticancer action of sodium selenite against peritoneal cancer in mice. Pharmacol. Res..

[60] Huang Y., Jia Z., Xu Y., Qin M., Feng S. (2020). Selenium protects against LPS-induced MC3T3-E1 cells apoptosis through modulation of microRNA-155 and PI3K/Akt signaling pathways. Genet. Mol. Biol..

[61] Torres S., Campos V., León C., Rodríguez-Llamazares S., Rojas S., Gonzalez M., Smith C., Mondaca M. (2012). Biosynthesis of selenium nanoparticles by Pantoea agglomerans and their antioxidant activity. J. Nanopart. Res..

[62] Bai Y., Qin B., Zhou Y., Wang Y., Wang Z., Zheng W. (2011). Preparation and antioxidant capacity of element selenium nanoparticles sol-gel compounds. J. Nanosci. Nanotechnol..

[63] Tang W., Li Y., Osimiri L., Zhang C. (2011). Osteoblast-specific transcription factor Osterix (Osx) is an upstream regulator of Satb2 during bone formation. J. Biol. Chem..

[64] Reible B., Schmidmaier G., Moghaddam A., Westhauser F. (2018). Insulin-like growth factor-1 as a possible alternative to bone morphogenetic protein-7 to induce osteogenic differentiation of human mesenchymal stem cells in vitro. Int. J. Mol. Sci..

[65] Reible B., Schmidmaier G., Prokscha M., Moghaddam A., Westhauser F. (2017). Continuous stimulation with differentiation factors is necessary to enhance osteogenic differentiation of human mesenchymal stem cells in-vitro. Growth Factors.

[66] Westhauser F., Karadjian M., Essers C., Senger A.-S., Hagmann S., Schmidmaier G., Moghaddam A. (2019). Osteogenic differentiation of mesenchymal stem cells is enhanced in a 45S5-supplemented β-TCP composite scaffold: An in-vitro comparison of Vitoss and Vitoss BA. PLoS ONE.

[67] Erkhembayar S., Mollbrink A., Eriksson L.C. (2012). The effect of sodium selenite on liver growth and thioredoxin reductase expression in regenerative and neoplastic liver cell proliferation. Biochem. Pharmacol..

[68] Kim J., Lee K.Y., Lee C.M. (2016). Selenium Nanoparticles Formed by Modulation of Carrageenan Enhance Osteogenic Differentiation of Mesenchymal Stem Cells. J. Nanosci. Nanotechnol..

[69] Zhang Y., Wang J., Zhang L. (2010). Creation of highly stable selenium nanoparticles capped with hyperbranched polysaccharide in water. Langmuir.

